# Fluid resuscitation with balanced crystalloids versus normal saline in critically ill patients: a systematic review and meta-analysis

**DOI:** 10.1186/s13049-022-01015-3

**Published:** 2022-04-18

**Authors:** Wei-Hua Dong, Wen-Qing Yan, Xin Song, Wen-Qiang Zhou, Zhi Chen

**Affiliations:** 1 Department of Emergency, Jiangxi Provincial People’s Hospital, 92 AiGuo St., Nanchang, 330006 Jiangxi China; 2 Medical Department, Nanchang University, Nanchang, Jiangxi China

**Keywords:** Balanced crystalloids, Saline, Intensive care unit, Meta-analysis, Trial sequential analysis

## Abstract

**Background:**

Intravenous fluids are used commonly for almost all intensive care unit (ICU) patients, especially for patients in need of resuscitation. The selection and use of resuscitation fluids may affect the outcomes of patients; however, the optimal resuscitative fluid remains controversial.

**Methods:**

We systematically searched PubMed, Embase, and CENTRAL. Studies comparing balanced crystalloids and normal saline in ICU patients were selected. We used the Cochrane Collaboration tool to assess the risk of bias in studies. The primary outcome was mortality at the longest follow-up. Secondary outcomes included the incidence of acute kidney injury (AKI) and new renal replacement therapy (RRT).

**Results:**

A total of 35,456 patients from eight studies were included. There was no significant difference between balanced crystalloid solutions and saline in mortality (risk ratio [RR]: 0.96; 95% confidence interval [*CI*]:0.92–1.01). The subgroup analysis with traumatic brain injury (TBI) showed lower mortality in patients receiving normal saline (RR:1.25; 95% CI 1.02–1.54). However, in patients with non-TBI, balanced crystalloid solutions achieved lower mortality than normal saline (RR: 0.94; 95% CI 0.90–0.99). There was no significant difference in moderate to severe AKI (RR: 0.96; 95% CI 0.90–1.01) or new RRT (RR: 0.94; 95% CI 0.84–1.04).

**Conclusions:**

Compared with normal saline, balanced crystalloids may not improve the outcomes of mortality, the incidence of AKI, and the use of RRT for critically ill patients. However, balanced crystalloids reduce the risk of death in patients with non-TBI but increase the risk of death in those with TBI. Large-scale rigorous randomized trials with better designs are needed, especially for specific patient populations.

**Supplementary Information:**

The online version contains supplementary material available at 10.1186/s13049-022-01015-3.

## Background

Fluid resuscitation is performed for patients in the intensive care unit (ICU) due to infection, shock, and burns [1, 2]. The selection and use of resuscitation fluids may affect the outcome of patients [3, 4]. Saline is the most widely used and readily available liquid in clinical practice. Despite being referred to as “normal” saline, it contains a higher chloride concentration and lacks bicarbonate than the plasma [5]. For the concerns that saline may increase the risk of acute kidney injury (AKI) [6, 7], clinicians may favor balanced crystalloids in critically ill patients requiring massive infusion. However, it is still unclear whether balanced crystalloids can improve the prognosis of critically ill patients [8, 9].

Preclinical studies showed that using saline may cause hyperchloremic metabolic acidosis, inflammation, hypotension, AKI, and death [2]. While there was no significant evidence that balanced crystalloids can reduce the risks of death and AKI in clinical randomized controlled studies (RCTs) [9–11], a meta-analysis [8] revealed that balanced crystalloids reduce the length of hospital stay, mortality, and incidence of AKI in critically ill patients. These results could hardly reflect the real mortality due to assignable heterogeneity and many confounding factors when merging observational studies. Subsequently, Zwager et al. [12] tried to include RCT studies for meta-analysis and sequential trial analysis. Their results showed that balanced crystalloids cannot reduce the risk of mortality and AKI in critical patients. Moreover, the results of sequential trail analysis did not cross the invalid boundary. Additionally, for inadequate samples, the current cumulative samples size cannot reach the desired size; although balanced crystalloids could reduce the mortality in the subgroup of sepsis patients, this evidence was low in quality.

Two large RCTs [13, 14] investigating the effects of balanced crystalloids and saline on the prognosis of critically ill patients in ICU have been published in recent years. We, therefore, hope to provide new evidence for the selection of resuscitation fluids for critically ill patients in the ICU through a rigorous systematic review and meta-analysis. A sequential analysis of the experiment must be conducted to determine whether the current cumulative sample size is enough to utilize medical resources and avoid waste adequately.

## Methods

The systematic review and meta-analysis were performed according to our protocol registered at the International Prospective Register of Systematic Reviews (PROSPERO; No. CRD42022304749) and followed the Preferred Reporting Items for Systematic Reviews and Meta-analyses statement [13].

### Search strategy

This meta-analysis searched PubMed, Embase, and CENTRAL databases from inception to 13 February 2022. The following keywords were used for the search: “balanced crystalloid solutions”, “saline solution”, “fluid management”, “intensive care units”, and “critically ill patients”. The search strategy for PubMed can be found in Additional file 1.

### Eligibly criteria

The inclusion criteria were as follows: (1) The trial was designed as a randomized controlled trial; (2) study subjects were critically ill patients (≥ 18 years old) requiring fluid resuscitation; (3) studies compared balanced solution and saline; and (4) the trial reported at least one of the outcomes (mortality, the incidence of AKI, and the incidence of new RRT).

The exclusion criteria were as follows: (1) fluids were used as maintenance rather than resuscitation, and (2) the study was a secondary analysis of the original data.

### Study selection

Two independent investigators performed the study selection. Disagreements between two investigators were resolved in meetings or adjudicated by a third reviewer.

### Data extraction

Two independent reviewers (DWH and YWQ) used a standardized form to perform the data extraction. The following data on study characteristics were collected: first author, publication year, sample size, mean age, severity, the cumulative volume of fluid, balanced crystalloid type, and follow-up time. The other two independent reviewers (ZWQ and SX) evaluated the data to ensure its accuracy. Two reviewers assessed the methodological quality of included trials according to the Cochrane Risk of Bias Tool.

### Risk of bias

Two authors (DWH, YWQ) independently assessed the study quality, study limitations, and the extent of potential bias using the Cochrane Collaboration’s risk of bias tool [14]. The following domains were assessed: random sequence generation, allocation concealment, blinding of participants and personnel, blinding of outcome assessments, incomplete outcome data, selective reporting, and other biases. Funnel plots for the primary outcomes were generated to assess publication bias.

### Statistical analyses

The statistical analysis was accomplished using the Cochrane systematic review software and Review Manager (RevMan; Version 5.3). Measurement data were expressed as means and standard deviations and 95% confidence intervals (95% CI). Enumeration data are expressed as risk ratios (RR) and 95% CI. Assessment of heterogeneity was completed using the chi-squared test. The *I*^*2*^ statistic was used for the determination of heterogeneity. The fixed-effect model was applied if low or moderate heterogeneity (*I*^*2*^ < 50%, *P* < 0.1). Otherwise, the random-effects model was used. Subgroup and sensitivity analyses were performed to investigate potential between-study heterogeneities and estimate other potentially confounding factors.

### Trial sequential analysis (TSA)

We assessed the risk of false positives or false negatives in the meta-analyses by TSA [15]. Sequential monitoring boundaries were established to limit the global type I error to 5%. Boundaries considered a power of 80% to detect a relative risk of a 5% decrease in mortality at the longest follow-up. The control group mortality for the various settings was selected (Mortality at the longest follow-up, mortality of septic patients, mortality of traumatic brain injury [TBI], mortality of non- TBI, the need for new renal replacement therapy [RRT], and moderate to severe AKI were set at 27.2%, 48.9%, 23%, 21.9%, 8%, and 28.4% respectively in the ICU). When the cumulative Z-curve enters the futility area or crosses the trial sequential monitoring boundary, the anticipated intervention effect may reach a sufficient level of evidence. If the Z-curve does not cross any of the boundaries and the required information size has not been reached, the evidence is rendered inadequate to conclude. The TSA was conducted using TSA version 0.9 beta (The Copenhagen Trial Unit, Centre for Clinical Intervention Research, Rigshospitalet, Copenhagen, Denmark), 2016.

## Results

### Study selection and study characteristics

The flow diagram showed the study selection process in Additional file 2: Fig S1. Eight randomized controlled trials [10, 11, 16–21], recruiting 35,456 patients, provided data for meta-analysis. We included one trial for which we could only source the abstract [20]. The main characteristics of the included studies are summarized in Table 1. All trials focused on patients in the ICU. Sample sizes ranged from 65 to 11,052. One trial [17] (46 patients) analysed 30-day mortality, two trials [11, 19] (16,776 patients) analysed the 60-day mortality, two trials [16, 21] (15,366 patients) analysed the 90-day mortality, and two trials [10, 18] (2329 patients) analysed the in-hospital mortality.

**Table 1:** Study characteristics

Study	Sample Size (n)	Age (years)	Men n (%)	Severity	Type of balanced saline	Serum creatinine	Sepsis patients n (%)	Invasive mechanical ventilation patients n (%)	Cumulative volume of fluids (at first 24 h) (ml)	Cumulative volume of fluids during follow-up period	Mortality: follow-up period in days
Finfer 2022	5037	BS: 61.7(16.4)^a^	BS: 1578(62.7)	APACHE II BS: 19.0 (14.0–26.0)^b^	Plasma-Lyte 148	BS: 127.4(109.8)^a^	BS: 1048(42.8)	BS: 1861(75.9)	BS: 1609^c^	BS: 3900 (2000–6700)^b^	90
		NS: 62.1(16.5)^a^	NS: 1511(59.9)	NS: 19.0 (14.0–25.0)^b^		NS: 125.9(112.0)^a^ (μmol/L)	NS: 1023(41.8)	NS: 1881(76.8)	NS: 1522^c^	NS: 3700(2000–6300)^b^	
Zampieri2021	11,052	BS: 60.9(17.0)^a^	BS: 2909(55.6)	APACHE II BS: 12 (8–17)^b^	Plasma-Lyte 148	BS: 1.2 (0.9)^a^	BS: 966 (18.5)	BS: 2304(44.2)	BS: 2078^c^	BS: 4100(2900)^a^	90
		NS: 61.2(16.9)^a^	NS: 2956(55.9)	NS: 12 (8–17)^b^		NS: 1.2 (0.9)^a^ (mg/dl)	NS: 1015(19.2)	NS: 2340(44.3)	NS: 2096^c^	NS: 4100(2900)^a^	
Young 2015	2278	BS: 60.1(16.8)^a^	BS: 739 (64)	APACHE II BS: 14.1(6.9)^a^	Plasma-Lyte 148	BS: 0.98 (0.76)^a^	BS: 41 (4)	BS: 768(67)	BS: 1226(653–2505)^b^	BS: 2000(1000–3500)^b^	In hospital
		NS: 61.0(16.3)^a^	NS: 746 (67)	NS: 14.1(6.7)^a^		NS: 0.99 (0.68)^a^ (mg/dl)	NS: 43 (4)	NS: 731 (66)	NS: 1431(784–2340)^b^	NS: 2000(1000–3300)^b^	
Young2014	65	BS: 38(19)^a^	BS: 16 (73)	ISS BS: 24(18)^a^	Plasma-Lyte A	BS: 1.0 (0.3)^a^	NR	NR	NR	BS: 10,300(2900)^a^	30
		NS: 39(14)^a^	NS: 19 (79)	NS: 2 22(14)^a^		NS: 1.0 (0.2)^a^ (mg/dl)				NS: 4100(6500)^a^	
Semler 2017	974	BS: 57 (42–68)^b^	BS: 268 (51.5)	NR	Lactated ringer’s solution and Plasma-Lyte A	BS: 0.83 (0.67–1.09^b^	BS: 130 (25.0)	BS: 174 (33.5)	BS: 1597^c^	BS: 1600(500–3600)^b^	60
		NS: 58 (46–71)^b^	NS: 246 (54.2)			NS: 0.86 (0.69–1.12)^b^ (mg/dl)	NS: 130 (28.6)	NS: 155 (34.1)	NS: 1238^c^	NS: 1400(500–3400)^b^	
Semler 2018	15,802	BS: 58(44–69)^b^	BS: 4540(57.2)	NR	Lactated ringer’s solution and Plasma-Lyte A	BS: 0.89(0.74–1.10)^b^	BS: 1167(14.7)	BS: 2723 (34.3)	BS: 1642^c^	BS: 1000(0–3210)^b^	60
		NS: 58(44–69)^b^	NS: 4557(58.0)			NS: 0.89(0.74–1.10)^b^ (mg/dl)	NS: 1169(14.9)	NS: 2731 (34.7)	NS: 1506^c^	NS: 1020(0–3500)^b^	
Verma 2016	67	BS: 62(45–70)^b^	BS: 21(63.6)	NR	Plasma-Lyte 148	BS: 85 (58–134)^b^	BS: 4 (12.1)	BS: 19 (57.6)	BS: 1090(620–2500)^b^	BS: 2900(1600–5600)^b^	In hospital
		NS: 64(46–72)^b^	NS: 21(61.8)			NS: 90 (60–121)^b^ (μmol/L)	NS: 3 (8.9)	NS: 19 (55.9)	NS: 1275(435–2243)^b^	NS: 3400(1200–5800)^b^	
Ratanarat 2017	181	NR	NR	NR	NR	NR	NR	NR	NR	NR	NR

APACHE, Acute Physiology and Chronic Health Evaluation; BS: Balanced saline; NS: Normal saline; NR, not report; ISS, Injury Severity Score; ^a^Mean ± standard error. ^b^Median (interquartile range). ^c^Mean

### Risk of bias in studies

The RCTs’ bias assessment was listed in Additional file 2: Fig. S2. Six trials were considered to have a low risk of bias, with adequate randomized sequences, concealed allocation, and analyzed outcomes of patients by the assigned group. Two trials were considered to have some concerns for the risk of bias. Ratanarat et al.’s study [20] was published as an abstract in a supplement. Although editors and peer reviewers evaluated the study design, there is an unknown risk of bias due to the lack of information. Enrolled patients were randomly assigned to the saline group balanced crystalloid group by month of admission to the ICU in Semler’s study [19]. This kind of randomization method could cause selection bias. The plot for mortality at the longest follow-up and incidence of AKI for studies was asymmetrical, implying that publication bias is strongly suspected. No publication bias was evident for the new RRT (Additional file 2: Fig. S3).

### Mortality at the longest follow-up

**Fig. 1:**
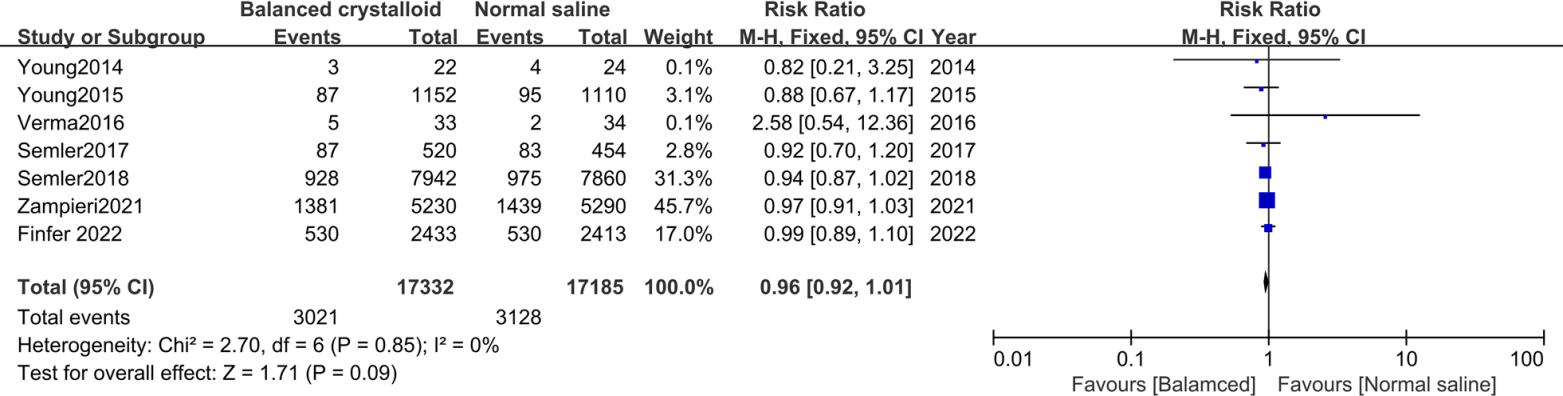
Forest plots for mortality at the longest follow-up for studies performed in ICU

**Fig. 2:**
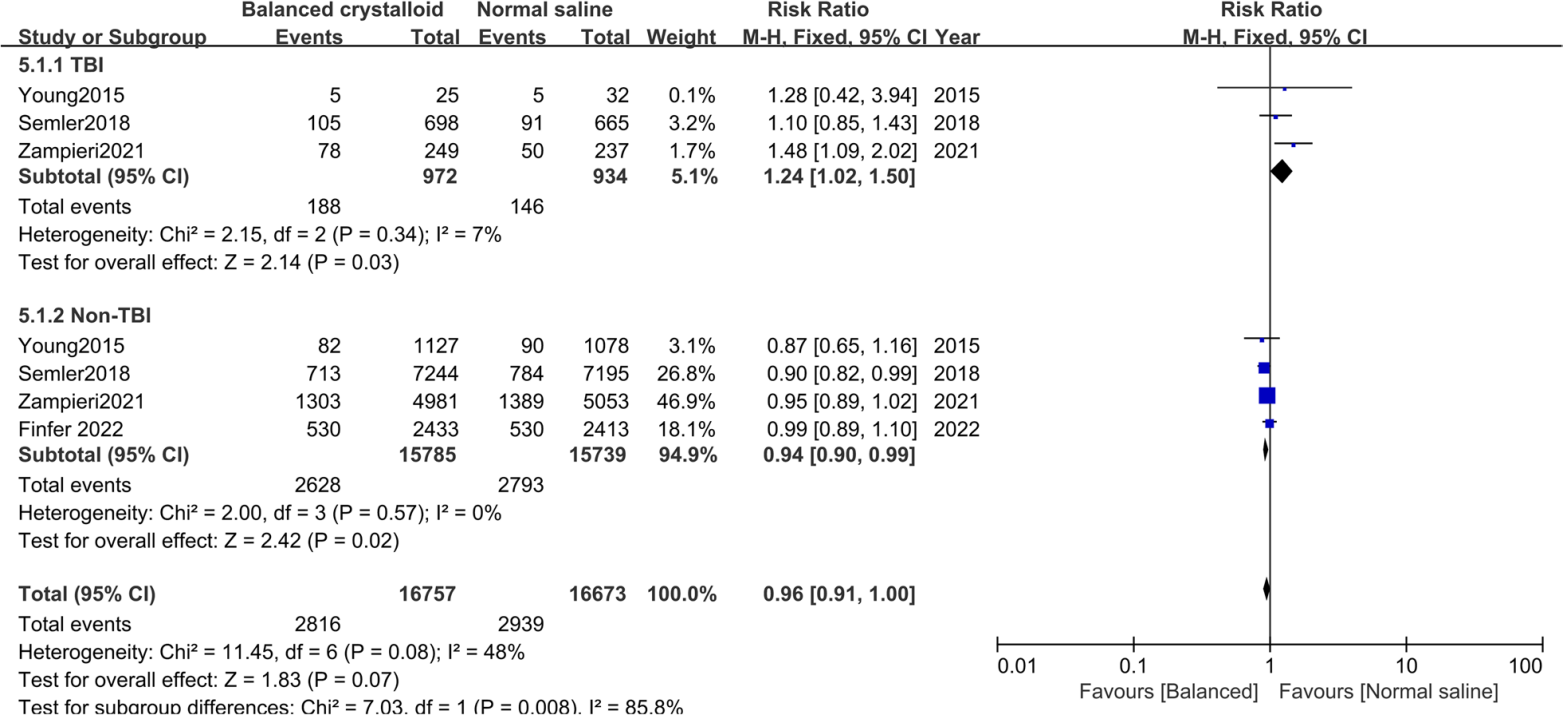
Forest plots of mortality for patients with TBI

Seven trials [10, 11, 16–19, 21] were included in the mortality analysis. There was no significant difference in patients receiving balanced crystalloid solutions versus normal saline (RR: 0.96; 95% CI 0.92–1.01) (Fig. 1). There was no significant heterogeneity (*P* = 0.09, *I*^*2*^ = 0%). Results from a subgroup analysis with sepsis (RR: 0.94; 95% CI 0.87–1.00; *I*^*2*^ = 0%) or non-sepsis (RR: 0.98; 95% CI 0.92–1.04; *I*^*2*^ = 0%) were similar to those in the over-all analysis (Additional file 2: Fig S4). The subgroup analysis with TBI showed lower mortality in patients receiving normal saline (RR: 1.24; 95% CI 1.02–1.50; *I*^*2*^ = 7%), whereas those non-TBI showed lower mortality in patients receiving balanced crystalloid (RR: 0.94; 95% CI 0.90–0.99; *I*^*2*^ = 0%) (Fig. 2).

### Renal outcomes

**Fig. 3:**
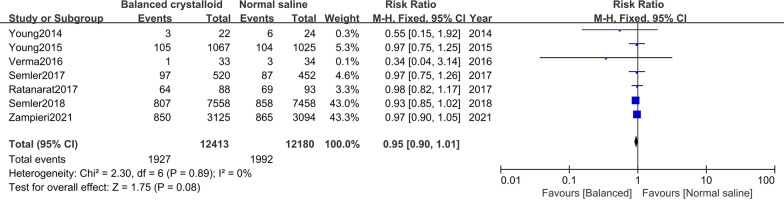
Forest plots for the development of moderate to severe acute kidney injury

**Fig. 4:**
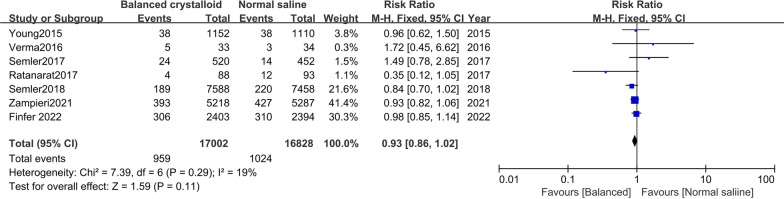
Forest plots for incidence of new RRT

Seven trials [10, 11, 16–20] were included in moderate to severe AKI analysis development. There was no significant difference in patients receiving balanced crystalloid solutions versus normal saline (RR: 0.95; 95% CI 0.90–1.01; *I*^*2*^ = 0%) (Fig. 3). Seven trials [10, 11, 16, 18–21] were included in the need for a new RRT analysis. We found no significant difference in the incidence of new RRT between patients receiving balanced crystalloid solutions compared with those receiving normal saline (RR: 0.93; 95% CI 0.86–1.02; *I*^*2*^ = 19%) (Fig. 4).

### Trial sequential analysis

**Fig. 5:**
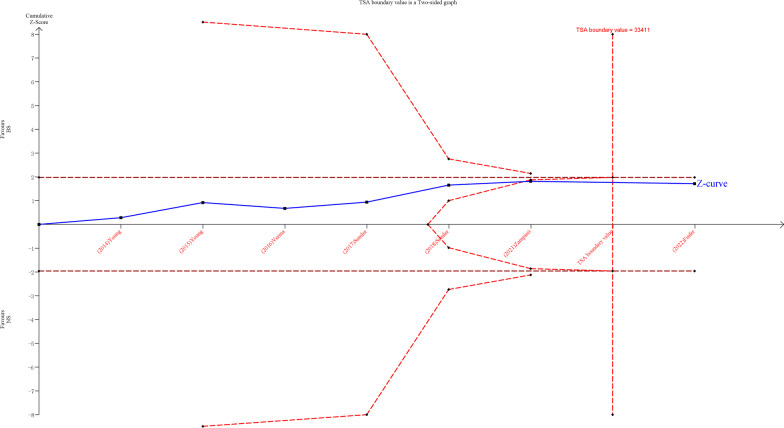
Trial sequential analysis for mortality at the longest follow-up. TSA used estimates of 27.2% for baseline mortality, 5% for relative risk reduction, 5% for alpha and 80% for power. The sample size reached required information size, but Z-curve not crossed conventional boundary and TSA boundary

**Fig. 6:**
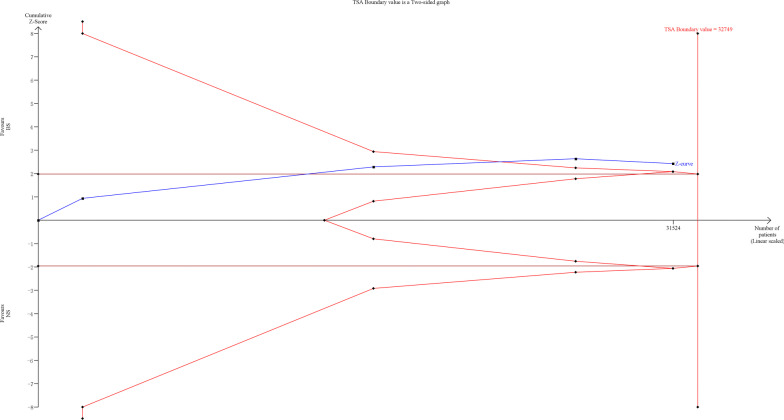
Trial sequential analysis for mortality of non-TBI patients. TSA used estimates of 21.9% for baseline mortality, 5% for relative risk reduction, 5% for alpha and 80% for power.The sample size not reached required information size, but Z-curve crossed conventional boundary and TSA boundary

Results of TSA for mortality at the latest follow-up can be found in Fig. 5. The Z-curve crossed trial sequential monitoring boundaries for futility and did not cross the conventional boundary. The sample size reached the required information size (n = 33,411) and did not require more trials for confirmation. However, the results of the TSA for mortality of patients with sepsis, the development of moderate to severe AKI, and incidence of new RRT showed that the Z-curve did not cross the conventional or trial sequential monitoring boundaries for benefit, harm, or futility (Additional file 2: Fig. S5). The mortality for patients with TBI was not available for TSA due to insufficient data. Results of TSA for mortality of non-TBI patients can be found in Fig. 6. The Z-curve crossed trial sequential monitoring boundaries and conventional boundaries for futility, indicating that although the sample size does not reach the required information size (n = 32,749), it has been proved that balanced crystalloids can reduce mortality in non-TBI patients.

## Discussion

In terms of this meta-analysis, there was no difference in mortality between the balanced crystalloids group and the normal saline group for adult ICU patients. The data crossed a conservative futility boundary, and further clinical trials may not alter the result. There was no significant difference in AKI incidence or the need for new RRT. However, balanced crystalloids reduce the risk of death in patients with non-TBI, which TSA confirmed. Balanced crystalloids may increase the risk of death in those with TBI, which needs further high-quality evidence to prove.

Fluid therapy is the cornerstone of treating critically ill patients in the ICU. The Surviving Sepsis Campaign guideline recommends crystal solution as the preferred resuscitation fluid; however, there is no recommendation on the types of crystal solution to administer as relevant research is lacking [22]. Some observational studies on operating rooms and ICUs suggested that hyperchloremia may be associated with higher mortality, including AKI risk. However, whether metabolic acidosis induced by saline affects organ function and mortality is uncertain. Indeed, humans can tolerate rather a significant acidosis (e.g., permissible hypercapnia in acute respiratory distress syndrome patients can reduce arterial blood PH to 7.23 without affecting organ function and mortality) [23]. Vasodilation induced by acidosis may aggravate critically ill patients’ shock [24]. However, fluid resuscitation is not performed alone in clinical practice. During intravenous fluid administration, critically ill patients undergo strict vital sign monitoring, including administration of vasoactive drugs, which to some extent reduce the effects of acidosis. The results of several prospective randomized controlled trials [10, 11, 16–19, 21] did not show that balanced crystalloids could reduce the mortality of critically ill patients. These supports reduce the effects of acidosis. After merging these studies, the results of the meta-analysis remained consistent. We correct the random error of mortality by TSA, and the results show that the sample size reached the required information size, which can be recognized as stable.

From a statistical standpoint, the mortality between the balanced crystalloids group and the normal saline group for critically ill patients was no difference in our meta-analysis. However, determining which critically ill patients would most likely benefit from balanced crystalloids is more meaningful for clinicians. Ostermann et al. [25] presented that understanding which ICU patient would most likely benefit from the use of a balanced electrolyte solution and which balanced solution would provide that benefit is important and understanding when the use of saline is equivalent or better. Hence, large-scale rigorous randomized trials with better designs are needed to provide robust evidence for clinical management, especially for specific patient populations. Evaluating which specific patient would most likely benefit from balanced crystalloids and which would benefit from normal saline is essential.

In the subgroup analyses, our study showed that balanced crystalloids might reduce the risk of death in patients with non-TBI but increase the risk of death in those with TBI. A possible explanation is that balanced crystalloids are a hypoosmotic solution, increasing the intracranial pressure in patients with TBI and increasing the incidence of hyponatremia [19]. Our results did support the option of administering normal saline to patients with TBI, which is clinically important. It is worth noticing that those with non-TBI showed lower mortality in patients receiving balanced crystalloid. And TSA was found that the finding of patients with non-TBI was reliable and conclusive. Subgroup analysis by patients with non-TBI included four trials [10, 16, 19, 21] with 33,430 patients. Our result for patients with non-TBI was utterly opposite to one RCT by Finfer et al. [21] published in 2022, which excluded patients with TBI or at risk for cerebral edema. The trial was prematurely terminated due to the coronavirus disease 2019 pandemic and included a reduction in the size of the recruitment target and unavailable data on the primary outcome. This trial was limited by its early termination. Furthermore, more than half the patients in the balanced crystalloids group received 500 ml or more saline. This may have attenuated a protective effect of balanced crystalloids. Hence, these findings provide necessary and reasonable suggestions between the fluid types for clinical management for critically ill patients with TBI or non-TBI.

In addition, previous retrospective study suggested [6, 26] that chloride-restrictive intravenous fluid administration strategy was associated with a significant decrease in AKI incidence and RRT use. However, in our study, we did not find that balanced crystalloids or saline can significantly decrease AKI incidence and use of RRT.

Our systematic review has several limitations. Firstly, although we sought to enroll critically ill patients requiring fluid resuscitation, some ICU patients receiving smaller infusion volumes were included in our study. Hence, large-scale rigorous randomized trials with better designs for fluid resuscitation are needed. Secondly, different studies reported different follow-up periods. Our primary outcome of all-cause mortality included in-hospital mortality, 30-day, 60-day, and 90-day mortality. However, heterogeneity was low in all-cause mortality for the included studies. Sensitivity analyses were also conducted by the sequential exclusion of each study and did not alter the results. Thus, it is reasonable to combine data from patients with different follow-up periods in our study. Thirdly, we included all critically ill patients in the ICU, and we could not determine if the results generalize to any specific population, such as ketoacidosis and sepsis. Finally, publication bias was possible, as demonstrated by a funnel plot, although efforts were made to conduct a thorough review of the literature.

## Conclusions

Compared with normal saline, balanced crystalloids may not improve the outcomes of mortality, the incidence of AKI, and the use of RRT for critically ill patients. However, balanced crystalloids reduce the risk of death in patients with non-TBI but increase the risk of death in those with TBI. Large-scale rigorous randomized trials with better designs are needed, especially for specific patient populations.

## Supplementary Information

**Additional file 1.** Search strategies for PubMed and Embase.

**Additional file 2.** **Figure S1.** Flow diagram illustrating the study selection process; **Figure S2.** Risk of bias summary; **Figure S3.** Funnel plots for mortality at the longest follow-up, incidence of AKI, and incidence of new RRT; **Figure S4.** Forest plots for mortality for patients with sepsis; **Figure S5.** Results of TSA for mortality for patients with sepsis, incidence of AKI, and incidence of new RRT.

## Data Availability

Not applicable.

## Abbreviations

ICU: Intensive care unit
AKI: Acute kidney injury
RRT: Renal replacement therapy
RCT: Randomized controlled studies
RR: Risk ratio
CI: Confidence interval
TBI: Traumatic brain injury
TSA: Sequential trial analysis
