# Supplementary material for: Fluid resuscitation with balanced crystalloids versus normal saline in critically ill patients: a systematic review and meta-analysis
Source: Scand J Trauma Resusc Emerg Med. 2022 Apr 18;30:28. doi: 10.1186/s13049-022-01015-3 (PMC9013977; doi:10.1186/s13049-022-01015-3)
Supplement: Supplementary file 1 — Additional file 1. Search strategies for PubMed and Embase. [file 13049_2022_1015_MOESM1_ESM.doc]

**PubMed**

1#. ((Ringer*) AND (lactat* OR acetate* OR solution*))

2#. ("crystalloid solutions" [Supplementary Concept]) OR (((balanced OR buffered)) AND (“crystalloid solution” OR “crystalloid solutions”))

3#. (“Plasma Lyte” OR Plasmalyte OR Plasma-Lyte OR Normosol OR Elo-Mel OR Elomel OR Sterofundin)

4#.(("Infusions, Intravenous"[Mesh] OR "Administration, Intravenous"[Mesh])) OR (Intravenous OR “fluid intervention” OR “fluid management” OR “fluid therapy”)

5#. ("Saline Solution, Hypertonic"[Mesh]) OR (Saline OR "sodium chloride" OR NaCl)

6#: ("Intensive Care Units" (MeSH) OR "Emergency Service, Hospital"[MeSH] OR "Critical Illness"[MeSH] OR "Intensive care" OR Emergency OR "Critical Illness" OR "Critically ill" OR "Critical care" OR ICU OR 24-hour*)

（1# OR 2# OR 3#）AND (#4 OR #5) AND #6

**Embase**

1#: 'balanced salt solution'/exp OR balance*:ti,kw OR buffer*:ti,kw OR 'buffer'/exp

2#: 'acetic acid plus gluconate sodium plus magnesium chloride plus potassium chloride plus sodium chloride'/exp OR 'plasma lyt*':ti,kw OR plasmalyt*:ti,kw OR ((low NEAR/3 chlorid*):ti,kw) OR ionolyt*:ti,kw OR jonolyt*:ti,kw OR sterofundin*:ti,kw OR isofundin*:ti,kw OR isolyte*:ti,kw OR normosol*:ti,kw OR plasmasol*:ti,kw OR ringerfundin*:ti,kw OR 'calcium chloride plus potassium chloride plus sodium chloride'/exp OR 'krebs ringer*':ti,ab,kw OR ringers:ti,ab,kw OR ringer:ti,ab,kw OR 'ringer s':ti,ab,kw OR hartmann*:ti,ab,kw OR hartman*:ti,ab,kw OR 'krh solution*':ti,ab,kw OR 'ars solution*':ti,ab,kw

3#: 'sodium chloride'/exp OR saline:ti,ab,kw OR nacl*:ti,ab,kw OR 'sodium chlorid*':ti,ab,kw OR salt:ti,ab,kw OR ((high NEAR/3 chlorid*):ti,ab,kw

4#'infusion fluid'/exp OR 'solution and solubility'/exp OR 'crystalloid'/exp OR 'intravenous drug administration'/exp OR infusion*:ti,kw OR infusat*:ti,kw OR intravenous*:ti,kw OR crystalloid*:ti,kw OR fluid*:ti,kw OR solution*:ti,kw OR 'sodium chloride'/exp OR saline:ti,kw OR nacl*:ti,kw OR 'sodium chlorid*':ti,kw

（1# OR 2#）AND (#3 OR #4)
