# Supplementary material for: Fluid resuscitation with balanced crystalloids versus normal saline in critically ill patients: a systematic review and meta-analysis
Source: Scand J Trauma Resusc Emerg Med. 2022 Apr 18;30:28. doi: 10.1186/s13049-022-01015-3 (PMC9013977; doi:10.1186/s13049-022-01015-3)
Supplement: Supplementary file 2 — Additional file 2. Figure S1. Flow diagram illustrating the study selection process; Figure S2. Risk of bias summary; Figure S3. Funnel plots for mortality at the longest follow-up, incidence of AKI, and incidence of new RRT; Figure S4. Forest plots for mortality for patients with sepsis; Figure S5. Results of TSA for mortality for patients with sepsis, incidence of AKI, and incidence of new RRT. [file 13049_2022_1015_MOESM2_ESM.doc]

**Electronic supplementary material**

This appendix has been provided to give readers additional information.

**Screening**

**Included**

**Eligibility**

**Identification**

Records identified through PubMed,Embase,CENTRAL searching (n=368)

Additional records identified through other sources
(n = 0)

Records after duplicates removed
(n = 195)

Full-text articles assessed for eligibility
(n = 18)

Full-text articles excluded, with reasons (n = 10)

Improper study design (n = 5)

No primary outcome (n = 1)

Secondary publication (n = 3)

Incorrect intervention (n = 1)

Studies included in qualitative synthesis
(n = 8)

Studies included in quantitative synthesis (meta-analysis)
(n = 8)

Studies excluded based on title and abstract
(n = 177)

Figure S1. Flow diagram illustrating the study selection process.


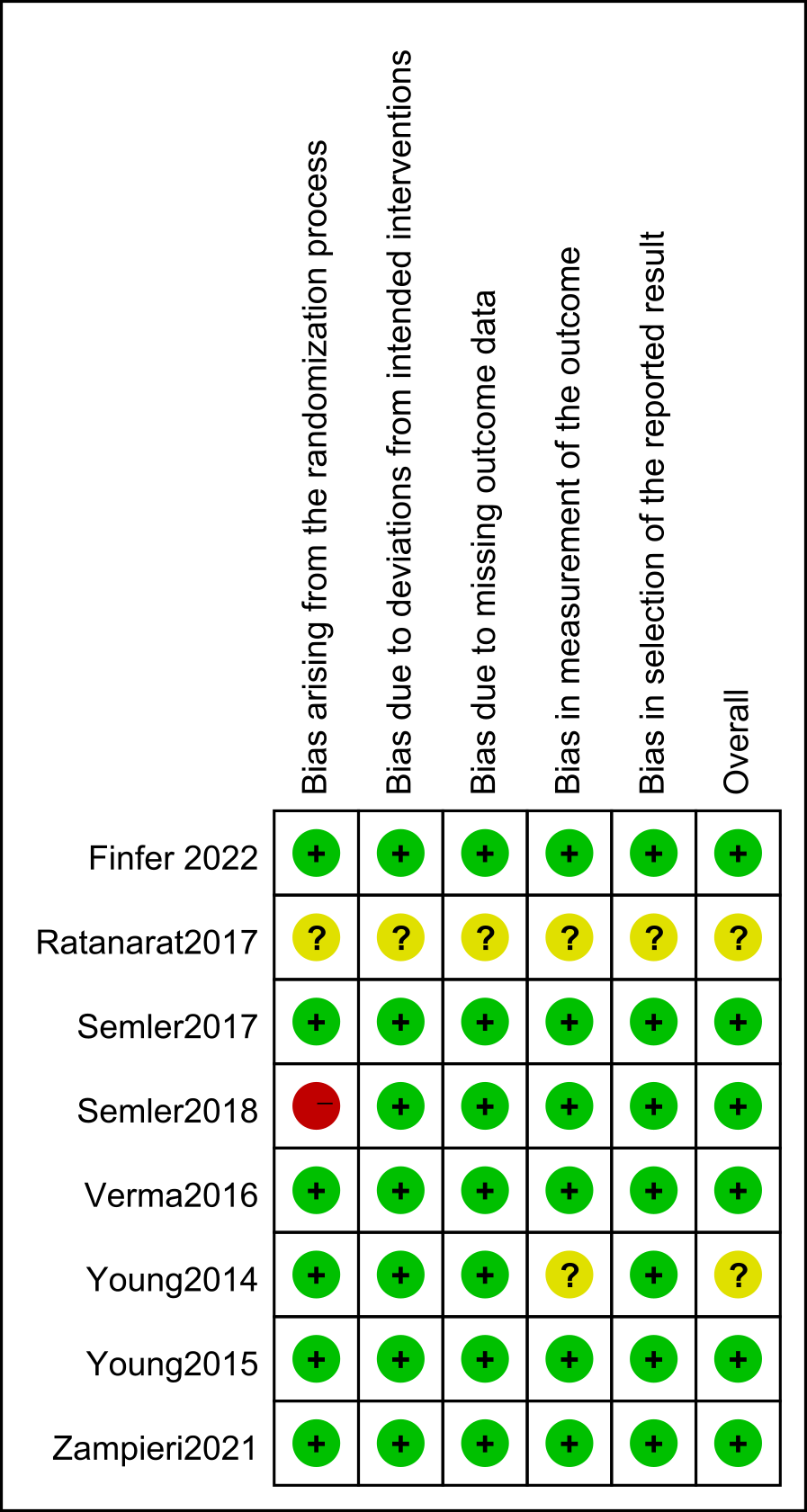


Figure S2. Risk of bias summary.

Figure S3 Funnel plots of included trials：


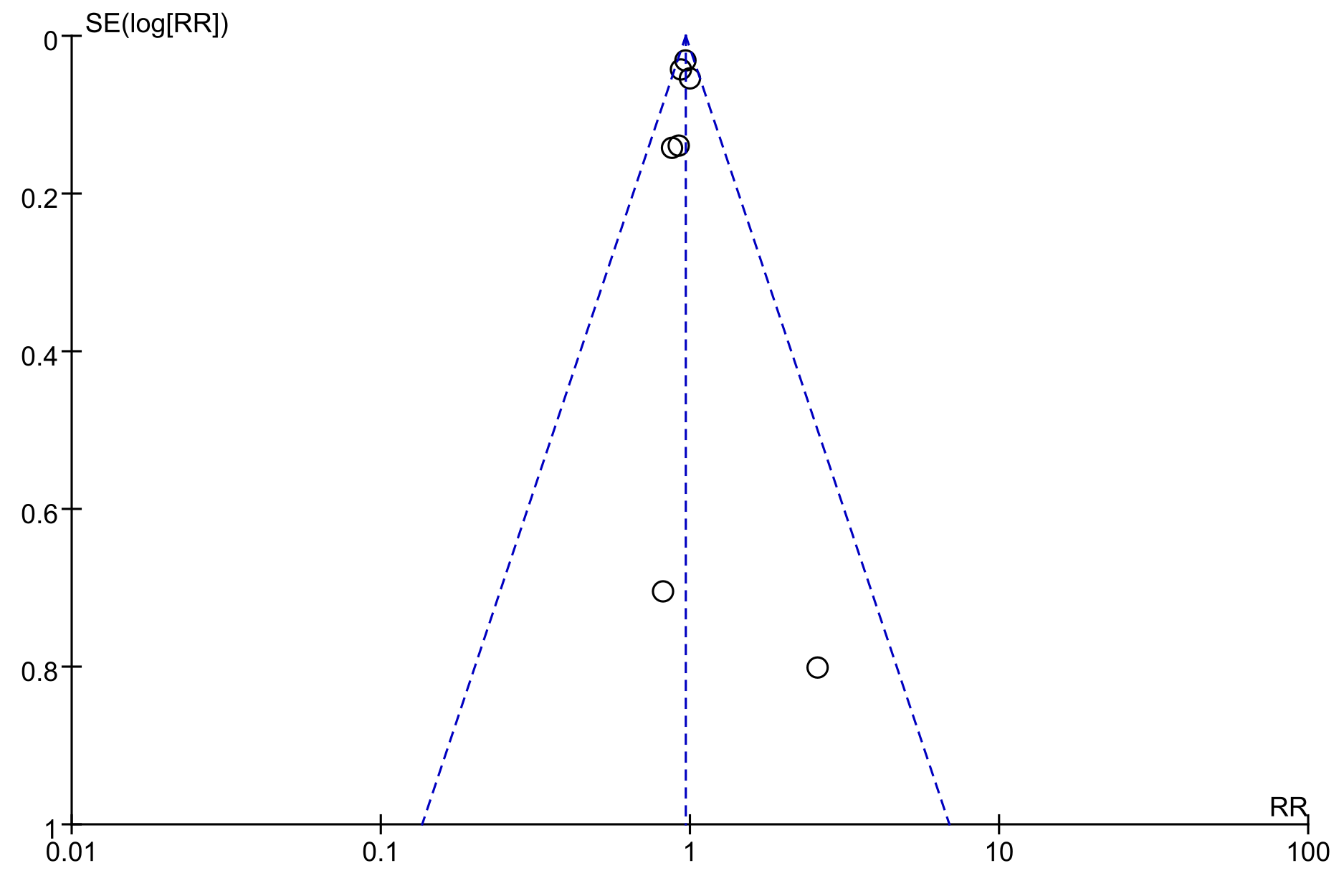


A: The plot for mortality at the longest follow-up


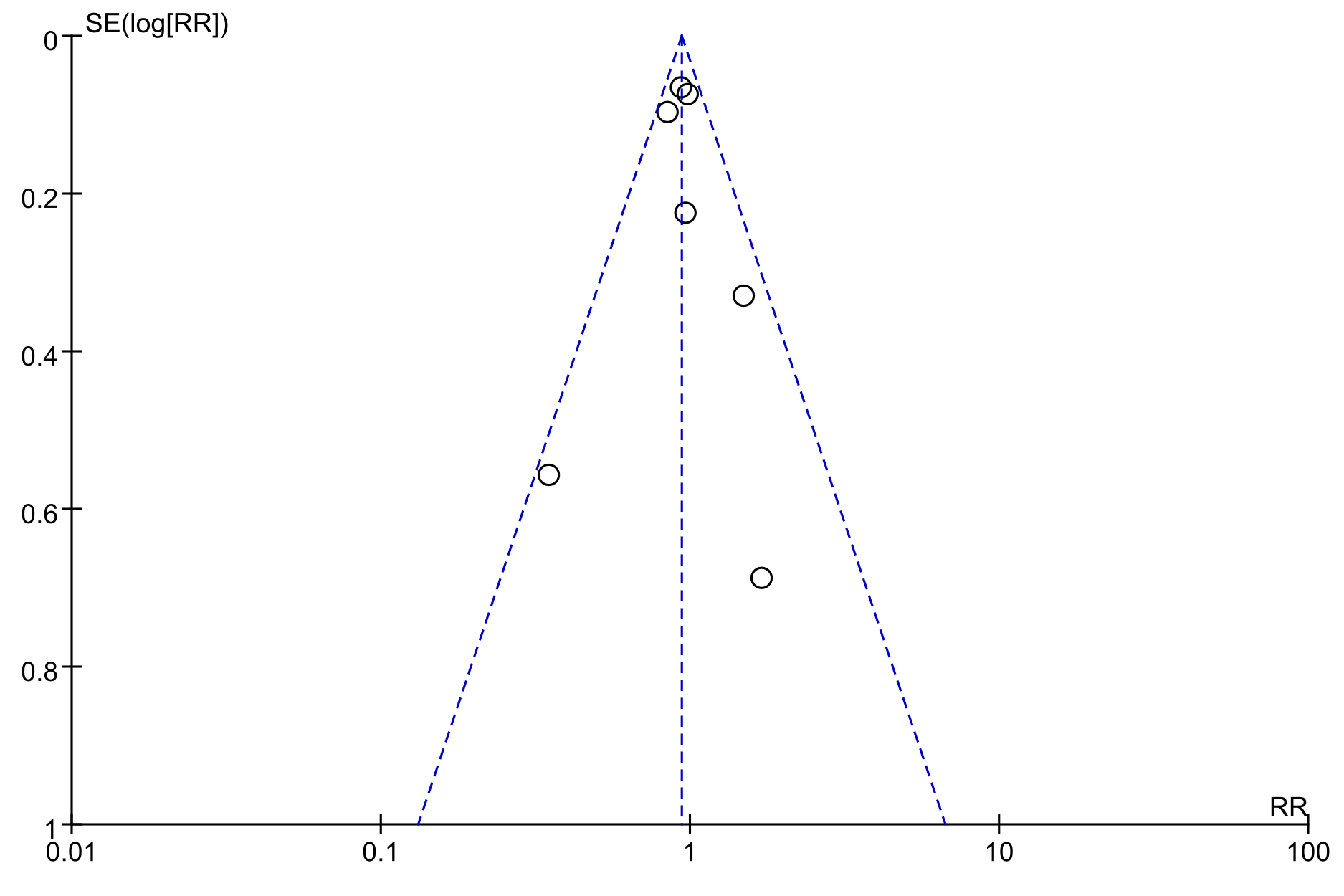


B：The plot for incidence of AKI


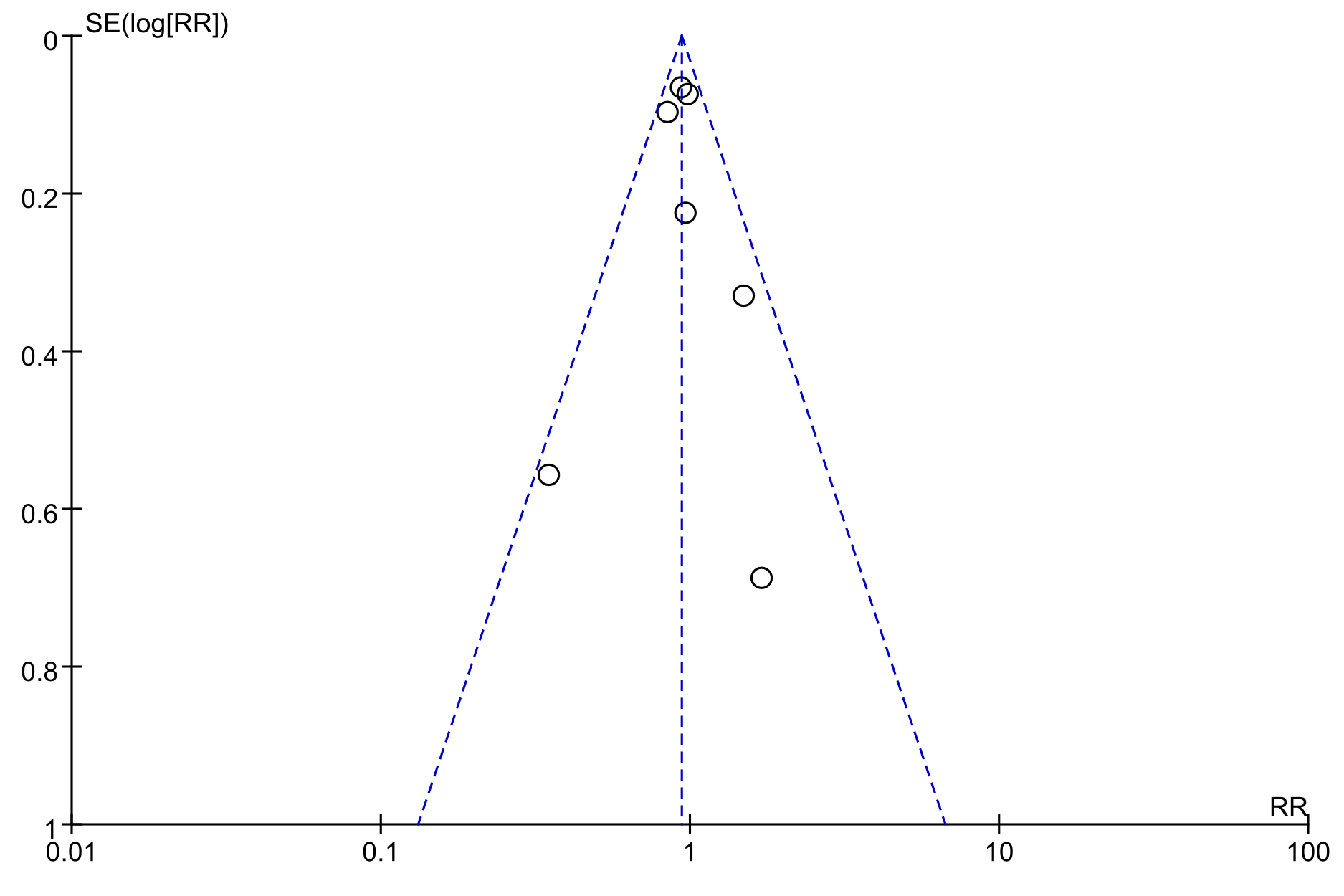


C: The plot for for new RRT


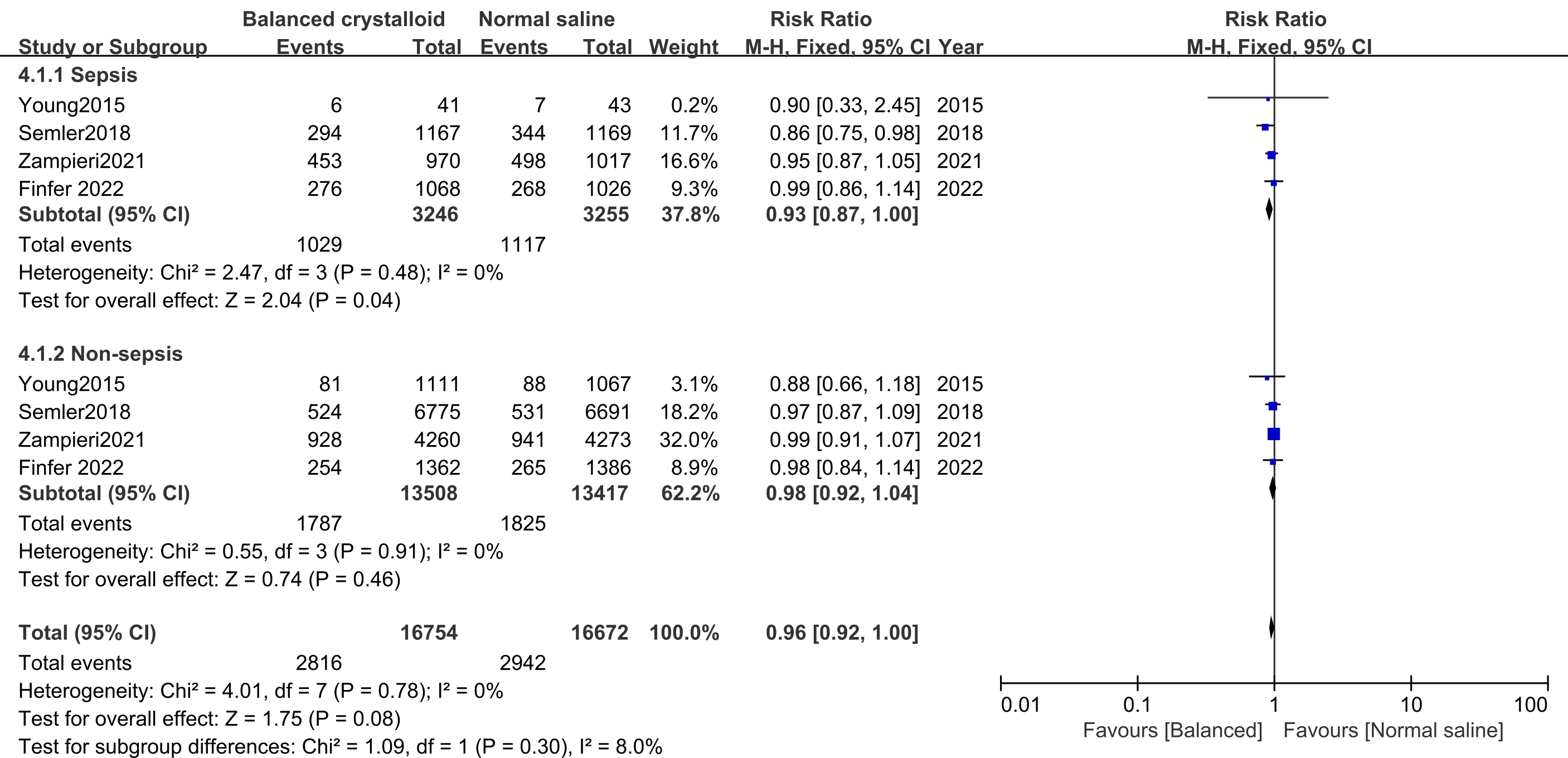


Figure S4. Forest plots for mortality for patients with sepsis.

Figure S5 Results of TSA


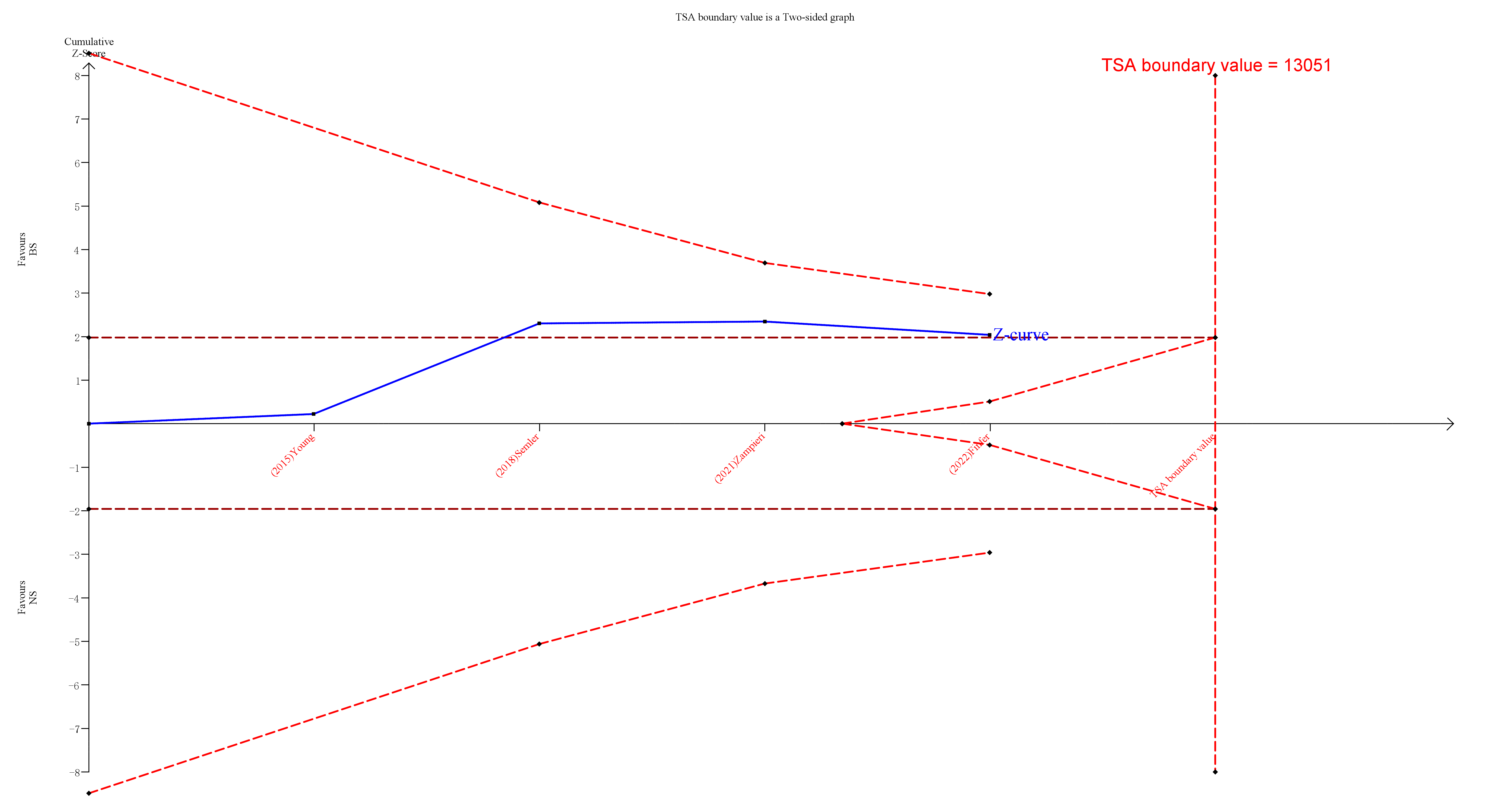


1. Results of TSA for mortality for patients with sepsis.


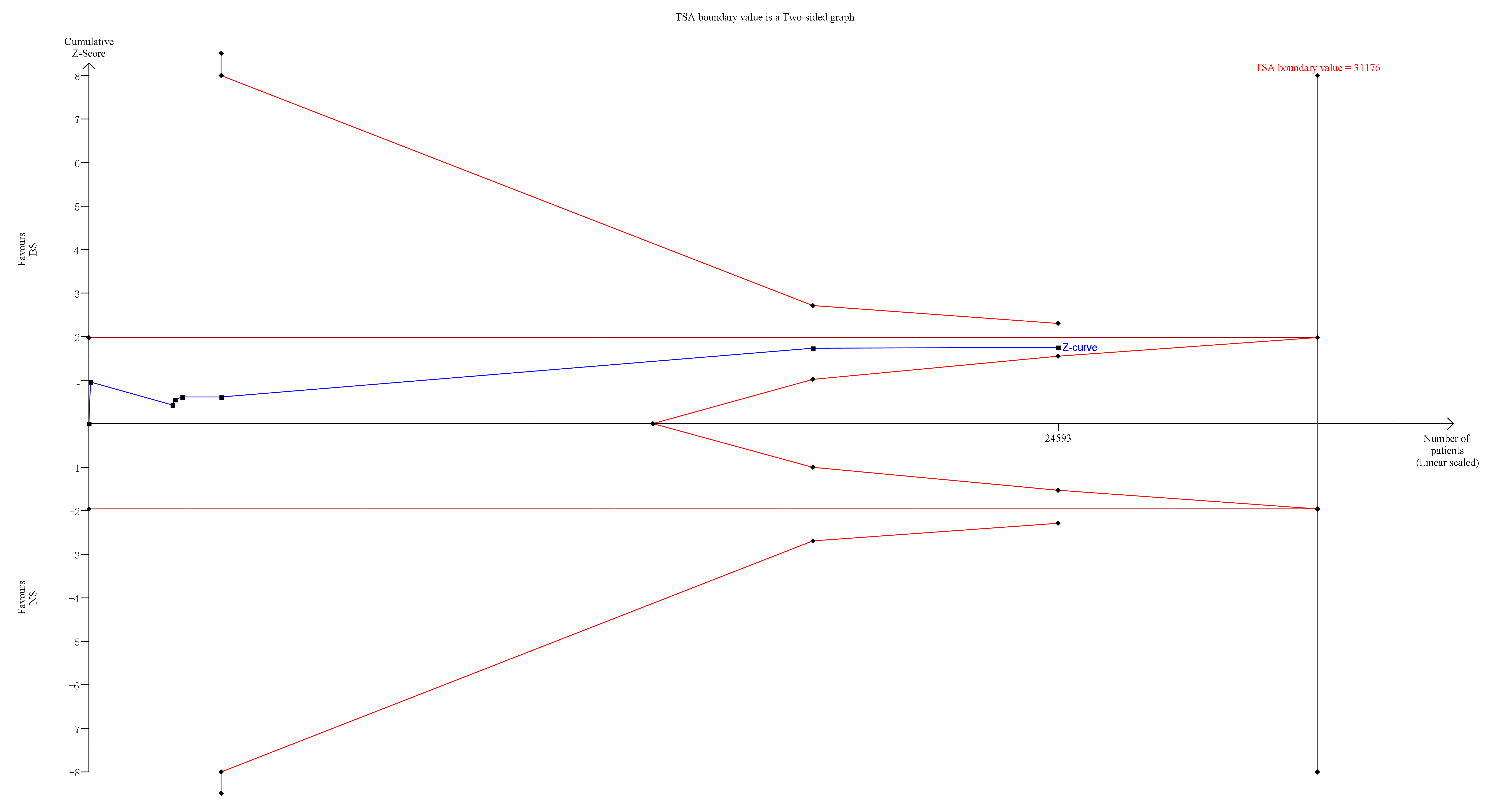


1. Results of TSA for the development of moderate to severe acute kidney injury


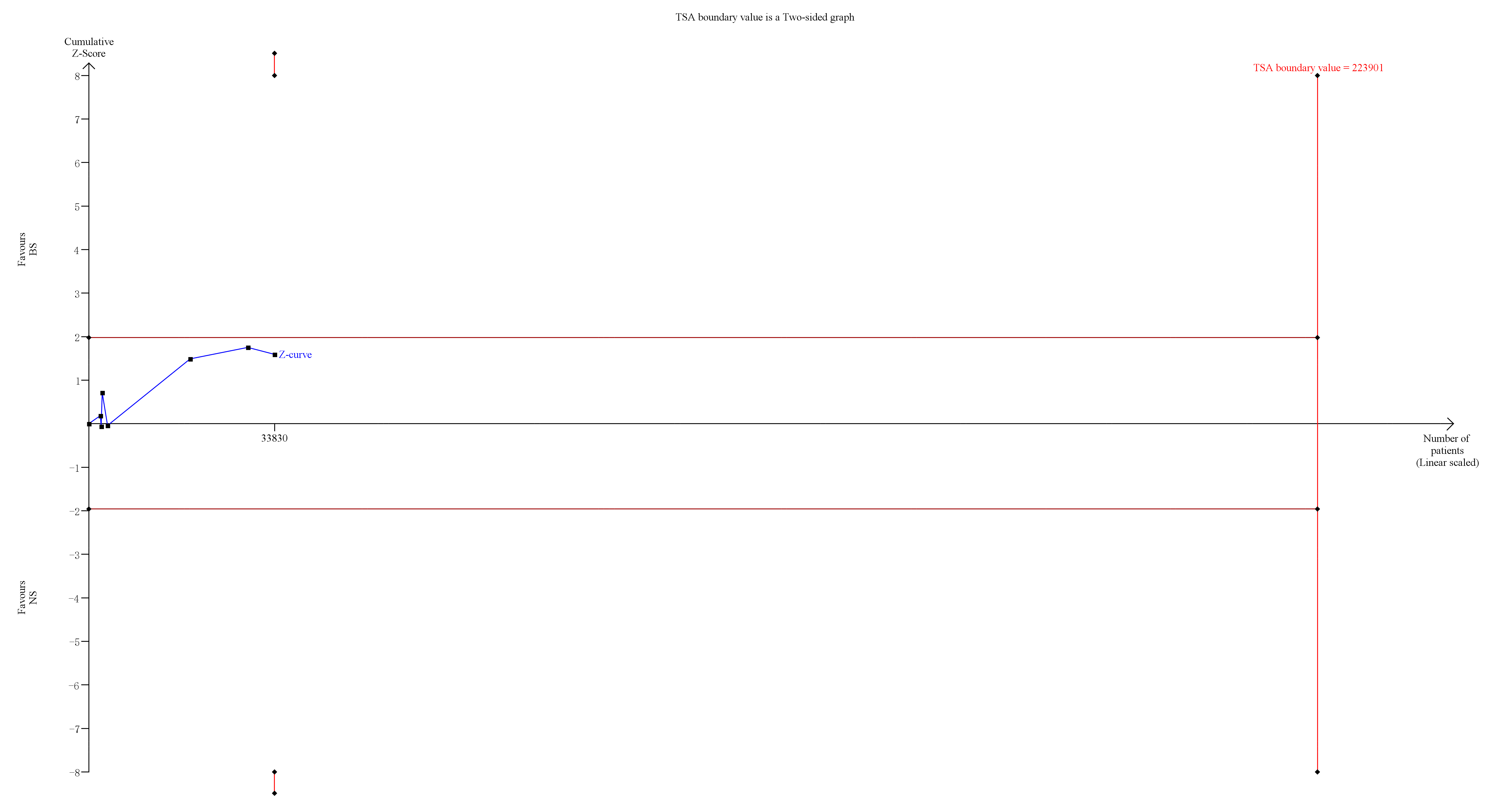


1. Results of TSA for incidence of new RRT
